# Extraction of Bioactive Metabolites from *Achillea millefolium* L. with Choline Chloride Based Natural Deep Eutectic Solvents: A Study of the Antioxidant and Antimicrobial Activity

**DOI:** 10.3390/antiox11040724

**Published:** 2022-04-06

**Authors:** Milena Ivanović, Dragana Grujić, Janez Cerar, Maša Islamčević Razboršek, Ljiljana Topalić-Trivunović, Aleksandar Savić, Drago Kočar, Mitja Kolar

**Affiliations:** 1 Faculty of Chemistry and Chemical Engineering, University of Maribor, Smetanova Ulica 17, SI-2000 Maribor, Slovenia; milena.ivanovic@um.si (M.I.); masa.islamcevic@um.si (M.I.R.); drago.kocar@fkkt.uni-lj.si (D.K.); 2 Faculty of Technology, University of Banja Luka, Vojvode Stepe Stepanovića 73, 78000 Banja Luka, Bosnia and Herzegovina; dragana.grujic@tf.unibl.org (D.G.); ljiljana.topalic-trivunovic@tf.unibl.org (L.T.-T.); aleksandar.savic@tf.unibl.org (A.S.); 3 Faculty of Chemistry and Chemical Technology, University of Ljubljana, Večna Pot 113, SI-1000 Ljubljana, Slovenia; janez.cerar@fkkt.uni-lj.si

**Keywords:** natural deep eutectic solvents (NADES), density and viscosity, yarrow, phenolic compounds, antioxidant activity, antimicrobial activity

## Abstract

In this study, the extraction efficiency of natural deep eutectic solvents (NADES) based on choline chloride as a hydrogen bond acceptor (HBA) and five different hydrogen bond donors (HBD; lactic acid, 1,4-butanediol, 1,2-propanediol, fructose and urea) was evaluated for the first time for the isolation of valuable bioactive compounds from *Achillea millefolium* L. The phytochemical profiles of NADES extracts obtained after ultrasound-assisted extraction were evaluated both spectrophotometrically (total phenolic content (TPC) and antioxidant assays) and chromatographically (UHPLC-MS and HPLC-UV). The results were compared with those obtained with 80% ethanol, 80% methanol, and water. The highest TPC value was found in the lactic acid-based NADES (ChCl-LA), which correlated with the highest antioxidant activity determined by the FRAP analysis. On the other hand, the highest antiradical potential against ABTS^+•^ was determined for urea-based NADES. Phenolic acids (chlorogenic acid and dicaffeoylquinic acid isomers), flavones (luteolin and apigenin), and their corresponding glucosides were determined as the dominant individual phenolic compounds in all extracts. The antibacterial and antifungal properties of the extracts obtained against four bacterial cultures and two yeasts were evaluated using two methods: the agar dilution method to obtain the minimum inhibitory concentration (MIC) and the minimum bactericidal or fungicidal concentration (MBC or MFC), and the disc diffusion method. ChCl-LA had the lowest MIC and MBC/MFC with respect to all microorganisms, with an MIC ranging from 0.05 mg mL^−1^ to 0.8 mg mL^−1^, while the water extract had the weakest inhibitory activity with MIC and MBC/MFC higher than 3.2 mg mL^−1^.

## 1. Introduction

The genus *Achillea* is one of the most economically and medicinally important genera in the family *Asteraceae*, with more than 100 different species growing in North America, different parts of Europe, East and Western Asia, Australia, New Zealand, and Middle East regions [1]. *Achillea millefolium* L. (yarrow), the best-known plant in this genus, is traditionally used in folk medicine for the treatment of lung (asthma and bronchitis), dyspepsia, and hepatobiliary disorders; skin inflammation; and headaches [2,3]. Previous research studies showed that *A. millefolium* L. has a disinfectant, anti-inflammatory, antispasmodic, anthelminthic, and antibacterial properties [1,4,5]. In addition, yarrow extracts are valued for their antioxidant activity, and have been shown to lower blood pressure, act as a diuretic, treat urinary tract infections, and relieve uterine and menstrual symptoms [1,6,7,8]. In vitro studies investigating the antimicrobial activity of alcoholic *A. millefolium* extracts revealed that the extracts were effective against several strains of bacteria and fungi, and in some cases were more potent than antibiotics from the penicillin family [9,10,11]. Based on the available literature data, the main secondary metabolites responsible for the biological activities of *A. millefolium* are essential oils and phenolic compounds [2]. Yarrow flowers contain 0.1% to 1% essential oil, and the main compounds identified are monoterpenes such as 1,8-cineole, sesquiterpenes such as farnesene and chamazulene, and lactones such as achillin [1,2,5]. On the other hand, the solvent extracts of the *A. millefolium* flower are rich in flavonoid glycosides such as apigenin-7-*O*-glucoside, luteolin-7-*O*-glucoside, quercetin-3-*O*-glucoside; flavonoids such as luteolin, apigenin, quercetin, and rutin; and some other phenolic compounds including chlorogenic acid and di-caffeoylquinic acid (DCQA) derivatives [6,7,8,9,12,13,14].

Various extraction methods have been used in order to extract these valuable bioactive compounds (in particular phenolic compounds) from yarrow. Solid–liquid extraction (maceration) or Soxhlet extraction with different solvents ranging from methanol, ethanol, alcohol–water mixtures to *N*-hexane, chloroform, and even sunflower oil are the most commonly described extraction procedures [6,12,14,15]. However, with the aim of reducing the use of large amounts of organic solvents and decreasing energy consumption and extraction time, modern extraction techniques such as ultrasound-assisted extraction (UAE) [3], microwave-assisted extraction (MAE) [16], and supercritical water extraction [7,17] have also been evaluated.

In recent years, in the extraction of bioactive compounds from natural sources, special attention has been paid to the development of clean, non-toxic, and biocompatible methods with improved extraction efficiencies using so-called deep eutectic solvents (DES) [18]. There are several reasons for this. First of all, DES, and especially their naturally occurring analogs, the natural deep eutectic solvents (NADES), are recognized as GRAS (generally recognized as safe) solvents [19]. In addition, the tunable physicochemical properties (viscosity, density, surface tension, and pH) of NADES, achieved by combining the appropriate hydrogen bond acceptors (HBA) and hydrogen bond donors (HBD), enable selective extraction, which is certainly one of their greatest advantages over conventionally used solvents [20]. Moreover, these physicochemical properties can be adjusted further by adding a suitable water content to the prepared DES/NADES. Finally, many authors have also reported the inhibitory effect of DES/NADES on various microorganisms [19,21]. In light of this, various DES/NADES have been developed and evaluated for the extraction of total phenolic compounds [22], as well as various classes of phenolic compounds: phenolic acids [23,24], flavonoids [25], anthocyanins [26,27], iridoids, and phenylpropanoids [28,29], among others [18,30], from plant material. The results obtained so far are promising, as most authors have report improved extraction efficiency, lower extraction costs, and low environmental impact. Moreover, the first experiments on direct application of DES/NADES extracts rich in bioactive compounds are described in recently published papers [31,32].

Thus, the aim of the present study was to evaluate different NADES extracts of *A. millefolium* obtained after ultrasound-assisted extraction (UAE). For this evaluation, UHPLC-MS and HPLC-UV analyses of the NADES extracts were used to monitor the concentrations of the main phenolic compounds, and to compare their contents with those of the extracts obtained with conventional solvents, such as 80% MeOH, 80% EtOH, and water. In addition, all of the extracts were analyzed spectrophotometrically for total phenolic content (TPC) and antioxidant activity using two different in vitro assays (ABTS and FRAP). The physicochemical properties of the synthesized NADES (viscosity and density) were determined, and correlations were predicted between the results and the determined extraction efficiencies. Although several studies have been conducted in the field of antimicrobial activity of yarrow extracts, to the best of our knowledge, this is the first report evaluating both the NADES solvent itself and NADES extracts of *A. millefolium* against pathogenic microorganisms such as *Staphylococcus aureus*, *Escherichia coli*, *Pseudomonas aeruginosa*, *Bacillus cereus*, and *Candida albicans*.

## 2. Materials and Methods

### 2.1. Chemicals and Samples

2,4,6-tris(2-pyridyl)-s-triazine (TPTZ reagent, ≥99%), 2,2′-azino-bis(3-ethylbenzothiazoline-6-sulfonic acid) diammonium salt (ABTS reagent), (±)-6-hydroxy-2,5,7,8-tetramethylchromane-2-carboxylic acid (trolox, 97%), choline chloride (≥98%), lactic acid (85%), 1,2-propanediol (99%), d-(-)-fructose (≥99%), urea (99–101%), potassium persulfate (K_2_S_2_O_8_), FeCl_3_·6H_2_O, and sodium acetate (CH_3_COONa), as well as the reference HPLC standards chlorogenic acid (>98.0%), 1,5-dicaffeoylquinic acid (1,5-DCQA, ≥97%), apigenin (≥99.0%), and luteolin (>98%), were purchased from Sigma-Aldrich (St. Louis, MO, USA). The standard of apigenin-7-*O*-glucoside was from Extrasynthèse (Genay, France). 1,4-butanediol (≥99%), Folin–Ciocalteu reagent (FCR), gallic acid (99%), acetic acid (99.8%), anhydrous sodium carbonate (Na_2_CO_3_), and FeSO_4_·7H_2_O were supplied by Merck (Darmstadt, Germany), while HPLC-grade methanol (MeOH, >99.9%), acetonitrile (>99.9%), and ethanol (EtOH, 96%) were supplied by Honeywell (Offenbach am Main, Germany).

The Mueller Hinton agar (MHA), Mueller Hinton broth (MHB), Nutrient agar (NA), and Sabouraud agar (SA) were purchased from Liofilchem (Roseto degli Abruzzi, Italy). Antibiotic discs: Erythromycin (15 µg), gentamicin (10 µg), ciprofloxacin (5 µg), and ampicillin (10 µg) were from the Mast Group (Bootle, UK), while nystatin (100 units) and fluconazole (100 µg) were obtained from Liofilchem (Roseto degli Abruzzi, Italy). The ultrapure water (resistance above 18.2 MΩ cm) was prepared fresh daily in the laboratory.

The plant material, the dried aerial parts of *A. millefolium*, were purchased from the specialized market “Prirodno bilje” d.o.o (Banja Luka, Bosnia and Herzegovina). According to the specification, the raw upper parts of the plant were collected at the full flowering stage and were air-dried for two weeks in a dark, ventilated place at room temperature (RT, 20–25 °C) and packed in paper bags. Before extraction, the sample was ground to a fine powder using a laboratory mill, and the average particle size was 0.75 mm. The obtained powder was collected in a paper bag and stored in a dry and dark place at room temperature until further use.

### 2.2. Preparation and Characterisation of the Natural Deep Eutectic Solvents (NADES)

A total of five different natural deep eutectic solvents (NADES; Table 1) were prepared using the standard heating and stirring method [23]. For this purpose, the components of the desired NADES were weighed into a round bottomed flask at the selected molar ratio (Table 1), and stirred over a water bath for at least 30 min. To reduce viscosity and facilitate handling, the prepared solvents were diluted further with 25% ultrapure water before use (NADES/water, 75%:25% *w*:*w*).

The densities (ρ, kg m^−3^) of these mixtures (NADES/water, 75%:25% *w*:*w*) were determined in the temperature range between 5 °C and 60 °C (278.15–333.15 K), in increments of 5 °C at an ambient pressure of 0.1 MPa using a DMA 5000 M vibrating tube densimeter (Anton Paar, Graz, Austria). The set temperatures were stable within the interval ± 0.005 °C, while the repeatability of the measurements was within ±5 × 10^−3^ kg m^−3^.

In addition, the dynamic viscosities (η, mPa·s) were measured in the same temperature range (from 5 °C to 60 °C) using a Lovis 2000 M/ME rolling-ball viscometer (Anton Paar, Austria). The diameter of the capillary used was 1.8 mm and was chosen according to the optimal run time of the measurements. Gilded rolling-balls were used to avoid corrosion of the rolling balls. The capillary was calibrated with the viscosity standard before use.

### 2.3. Ultrasound-Assisted Extraction (UAE) of Bioactive Compounds

For the extraction of the bioactive compounds of *A. millefolium*, UAE was applied [23]. Briefly, 500 mg of the ground and homogenized sample was weighed into a 50 mL conical centrifuge tube. Then, 10 mL of the selected NADES (NADES/water, 75%:25% *w*:*w*) was added and the extraction was performed in an ultrasonic bath (Vevor, Shanghai, China, ultrasonic frequency −40 kHz, ultrasonic power −480 W, and heating power −600 W) for 30 min at an elevated temperature (50 ± 1 °C). Subsequently, the supernatant, obtained by centrifugation at 10,000 rpm for 15 min using an Eppendorf-5804 R (Hamburg, Germany) laboratory centrifuge, was transferred into a 25 mL volumetric flask. To the residue, 10 mL of the fresh solvent was added, and the UAE was repeated under the same conditions (30 min at the elevated temperature 50 ± 1 °C). The supernatant obtained after the second centrifugation cycle (at 10,000 rpm for 15 min) was combined with the first supernatant, and the 25 mL flask was pooled to the mark with the corresponding NADES. In parallel, extraction with the conventionally used solvents (80% MeOH, 80% EtOH, and ultrapure water) was performed in the same way. All of the extractions were done in duplicate. Prior to instrumental analysis (either chromatographic or spectrophotometric), all extracts were diluted in the appropriate volume ratio with 80% MeOH and were filtered using a 0.20 μm polytetrafluoroethylene (PTFE) filter.

### 2.4. UHPLC-MS and HPLC-UV Identification and Quantification of Individual Compounds

Identification of individual phenolic compounds from the *A. millefolium* sample was performed on the methanolic extract using an ultra high-performance liquid chromatographic system (UHPLC, Thermo Scientific™ Vanquish™ Flex, Waltham, MA, USA) coupled to a triple quadrupole mass spectrometer (MS; Thermo Scientific™ TSQ Quantis™). The compounds were separated on an Eclipse XDB-C18 column (150 mm × 4.6 mm i.d, 5 µm) operated at RT, with an injection volume of 10 µL and a mobile phase flow rate of 1 mL min^−1^. The mobile phase consisted of (A) acetonitrile and (B) a 1% aqueous solution of acetic acid, and the gradient program was set as follows: 0–1 min 95% B, 1–18 min 74% B, 18–28 min 68% B, 28–40 min 59% B, 40–40.10 min 95% B, and 7 min re-equilibration of the column to the initial conditions. The total run time was 47 min. A heated electrospray probe in negative mode was used, and the following ionization parameters were applied: Capillary voltage, 3000 V; ion transfer tube temperature, 350 °C; sheath gas, 60 a.u. (arbitrary units); auxiliary gas, 10 a.u.; sweep gas, 2 a.u.; vaporiser temperature, 400 °C [23]. MS detection was performed considering a mass range of 80–700 *m*/*z*.

Quantification of the major phenolic compounds identified in all *A. millefolium* extracts was performed using an HPLC-UV chromatography system (Varian, Crowley, UK) equipped with a binary solvent pump (ProStar 210), a UV/VIS detector (ProStar 310), and an auto-sampler (ProStar 410). The same chromatographic column and chromatographic conditions were used as for the UHPLC-MS identification, while the detection wavelength was set to 325 nm. In addition, the calibration curves of the five commercially available standards at six concentration levels (from LOQ to 50 mg L^−1^) were constructed (linear regression line; R^2^): Chlorogenic acid (Y = 10143x + 3685.5; R^2^ = 0.9985), 1,5-dicaffeoylquinic acid (1,5-DCQA; Y = 8764x − 201.5; R^2^ = 0.9983), apigenin-7-*O*-glucoside (Y = 9500.5x + 2516.7; R^2^ = 0.9972), luteolin (Y = 16342x + 9187.6; R^2^ = 0.9981), and apigenin (Y = 14602x + 7903.1; R^2^ = 0.9981). The results were expressed as mg of each compound per gram dry weight of the *A. millefolium* sample (mg g^−1^ DW).

### 2.5. Spectrophotometric Measurements

The total phenolic content (TPC) of the extracts obtained was determined by the standard Folin–Ciocalteu spectrophotometric method [33], with some modifications, described in detail in our previous work [20]. The final TPC content was expressed as milligrams of gallic acid equivalent per gram of dry weight of the *A. millefolium* sample (mg GA g^−1^ DW).

The antioxidant activity of the extracts, expressed as milligrams of trolox equivalent per gram of *A. millefolium* dry weight (mg TE g^−1^ DW), was determined using the ABTS radical scavenging method described previously [34], with some improvements [35].

The antioxidant activity of *A. millefolium* extracts was also determined using the standard ferric reducing antioxidant power (FRAP) assay [36], with some modifications [35]. The final results were expressed as milligrams Fe^2+^ ion equivalent per gram dry weight (mg Fe^2+^ g^−1^ DW).

A Varian, Cary 50 Bio spectrophotometer was used for all of the spectrophotometric determinations.

### 2.6. Antimicrobial Activity of A. millefolium Extracts

In this study, two methods were used to determine the antimicrobial activity of *A. millefolium* extracts: the agar dilution method to obtain the minimum inhibitory (MIC) and minimum bactericidal or fungicidal (MBC or MFC) concentrations [37], and the disk diffusion method [38].

#### 2.6.1. Microorganisms and Preparation of the Inoculum

Four bacterial cultures, *Escherichia coli* ATCC 25922, *Staphylococcus aureus* ATCC 25923, *Pseudomonas aeruginosa* ATCC 10145, and *Bacillus cereus* ATCC 7004, and two yeasts, *Candida albicans* ATCC 10231 and *Candida albicans* isolate (clinically isolated), were used from the collection of the Laboratory of Bacteriology, Mycology, and Parasitology of the Veterinary Institute of the Republic of Srpska (“Dr. Vaso Butozan” Banja Luka, Bosnia and Herzegovina). For this study, bacterial and yeast cultures were prepared from the logarithmic phase and by direct colony suspension [37,38].

A standard inoculum of *Escherichia coli*, *Pseudomonas aeruginosa*, and *Bacillus cereus* from the logarithmic growth phase was prepared in the following way. Namely, the cultures were seeded on agar plates (MHA) and incubated at 37 °C for 24 h. After that, 3–5 isolated colonies were transferred to a 5 mL tube containing MHB and incubated for the next 2–6 h. Care was taken to ensure that the same incubation time of the culture in MHB occurred in each experiment.

On the other hand, *Staphylococcus aureus* was inoculated from agar slants onto NA, while the yeast cultures (*Candida albicans* ATCC 10231 and isolated *Candida albicans*) were inoculated from agar slants onto the SA. The prepared agar plates were then incubated for 24 h at 37 °C for *Staphylococcus aureus* and at 30 °C for yeasts. Thereafter, two or three colonies were collected directly into the MHB. After incubation for 2–6 h or after preparation of the colony suspension, the culture density was determined spectrophotometrically (at 620 nm), using the 0.5 McFarland standard (1.5 × 10^8^ cfu mL^−1^) for comparison. The cultures were then diluted properly and their density was adjusted to 1.5 × 10^6^ cfu mL^−1^.

#### 2.6.2. Agar Dilution Method

A series of dilutions in agar was prepared by adding an appropriate amount of the extract to the dissolved medium (MHA for bacteria and SA for yeasts) cooled to 45 °C. The final concentration of the extract in the medium was in the range of 1.2–3.2 mg mL^−1^ (1.2, 1.6, 2, 2.4, 2.8, and 3.2 mg mL^−1^). Then, the substrates were shaken well, and poured into sterile Petri dishes. After the medium had hardened, the cultures were seeded in 10 μL drops on the surface of the agar plates and incubated at 37 °C (bacteria) and 30 °C (yeasts) for 24 h. The inhibition zones of the growth of the microbial cultures were measured. The highest dilution of the tested extract that inhibited the visible growth of bacteria and yeasts was considered as the MIC value. From the plates that showed no visible signs of growth/cloudiness in the MIC determination, the test microorganisms were inoculated onto sterile MHA or SA plates. The plates were then incubated at 37 °C for bacteria and at 30 °C for yeasts, for 24 h. The lowest concentration that showed no growth of the test organisms was considered as MBC or MFC. The medium without extract was used as a positive control, while solvents (MeOH, EtOH, ChCl-But, ChCl-Prop, ChCl-Fruc, and ChCl-U) in the concentration range from 4% to 16% (4%, 6%, 8%, 10%, 12%, 14%, and 16%) were used as a negative control. On the other hand, ChCl-LA showed a strong antimicrobial activity, and was tested in the concentration range of 0.125%–8% (0.125%, 0.25%, 0.50%, 1%, 2%, 4%, 6%, and 8%). The positive controls were antibiotic disks of erythromycin (15 µg), gentamicin (10 µg), ciprofloxacin (5 µg), and ampicillin (10 µg) for the bacteria, and nystatin (100 units) and fluconazole (100 µg) for the yeasts.

#### 2.6.3. Disc Diffusion Method

From the prepared suspension, cultures were seeded onto sterile MHA (bacteria) and SA (yeasts) agar plates using a sterile cotton swab. Namely, 30 μL of a specific dilution of the extracts was applied to sterile antibiogram disks with a diameter of 6 mm (Filtres Fioroni, France) in an empty sterile Petri dish. After the disks had absorbed the extract, they were transferred carefully to the surface of the agar plate containing the seeded cultures and were pressed down lightly. The disk cultures were then incubated for 24 h at 37 °C (bacteria) and 30 °C (yeasts), and the zones of inhibition around the disks were measured after incubation.

### 2.7. Statistical Analysis

All of the extractions in this study were performed in duplicate, while instrumental measurements were performed in triplicate. The final results were reported as mean value ± standard deviation. For the statistical comparisons of the obtained results, the one-way ANOVA test was applied, followed by the Student–Newman–Keuls (S-N-K) post-hoc (at the 95% confidence level) using SPSS software: IBM SPSS Statistics for Windows, version 22.0 (IBM Corp., Armonk, NY, USA).

## 3. Results and Discussion

### 3.1. Characterisation of NADES; Density and Viscosity Measurements

In this study, five different NADES, based on choline chloride as the HBA and lactic acid, 1,2-propanediol, 1,4-butanediol, fructose, and urea as the HBD, were investigated as extraction media for the isolation of bioactive compounds from *A. millefolium* flowers (Table 1). All of the prepared solvents were highly viscous, transparent, and colorless liquids. As previously reported, the extraction potential of NADES is determined largely by both the chemical structures of the target compounds and the properties of the desired NADES, particularly density, viscosity, and pH [18,20,39]. Therefore, densities and viscosities in the temperature range 5–60 °C (278.15–333.15 K) were investigated in the first part of this work.

**Figure 1:**
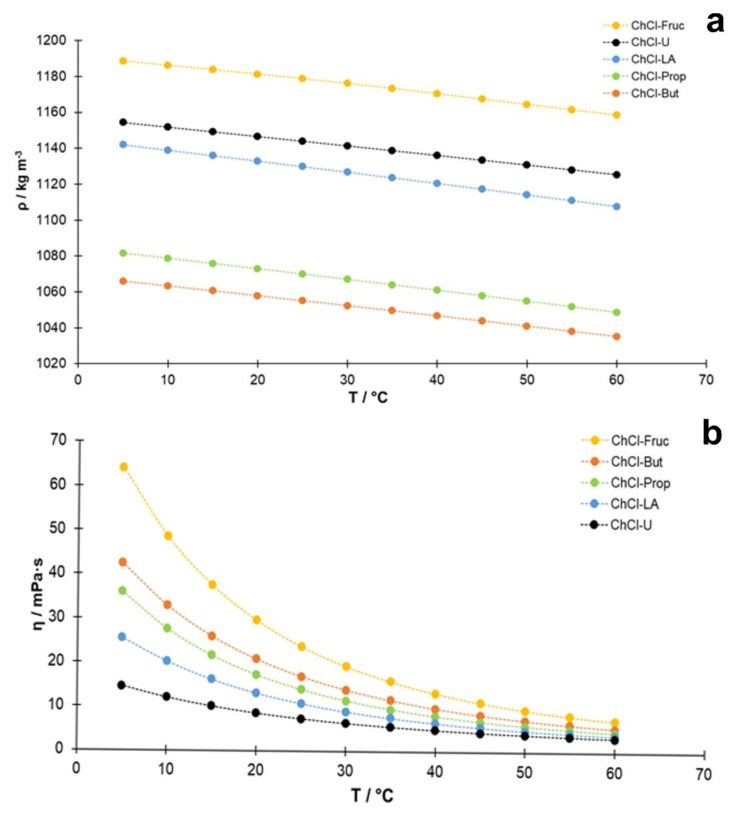
Temperature dependence of experimentally determined (**a**) density (ρ; kg m^−3^) and (**b**) viscosity (η; mPa·s) of NADES with 25% (*w*/*w*) water content.

In general, based on the results (Figure 1a), the density of the studied NADES decreased with increasing temperature, and this decrease was approximately linear with the temperature. Regarding the accuracy of the measured densities, the obtained values may be verified for the system reline/water containing 25% (*w*/*w*) water, where a good consistency was observed with the published data [40]. A second degree polynomial was usually used to describe the temperature dependence of viscosity in similar systems [40,41]. As can be seen in Figure 1a, the quadratic equation also describes this dependence well in this study. Another feature evident from Figure 1a is that the slope is quite similar for all the studied NADES, which can be explained by insignificant changes in the structural arrangement of NADES in the selected temperature range. In addition, comparing the densities of these NADES at 25 °C (298.15 K) with the densities of aqueous solutions of the components used as HBD [42,43], which have the same mass concentrations as in the NADES systems, some correlation can be found (Figure S1, Supplementary Materials). The data for the ChCl-Fruc system are not included in this comparison because of the different molar ratio between HBA and HBD (choline chloride/fructose, 2:1) than in the other cases (HBA/HBD, 1:2). Although the correlation in Figure S1 is not ideal, it shows that the main causes of the density differences between the studied NADES are the molar masses and volumes of the hydrogen bond donor molecules, while the contribution of other factors is smaller. It should also be noted that lactic acid cannot be obtained in pure form. because an equilibrium exists between the monomeric acid and the polylactic acid [43]. It is expected that the involvement of lactic acid in the formation of hydrogen bonds will have a significant effect on the equilibrium composition of the lactic acid components, and, hence, on the density of this system.

The measured dynamic viscosities, η (*T*), of the prepared NADES in the temperature range between 5 °C and 60 °C in increments of 5 °C are shown graphically in Figure 1b. The lowest viscosity at 50 °C (the temperature used in this study during the extraction process) was determined for ChCl-U (3.87 mPa·s), followed closely by lactic acid-based NADES (ChCl-LA, 4.24 mPa·s), while the highest value was detected for the sugar-based NADES (ChCl-Fruc, 9.50 mPa·s). In the case of polyalcohol-based NADES, a lower value of dynamic viscosity was observed for the ChCl-Prop (5.82 mPa·s) compared to the value of 7.11 mPa·s obtained for ChCl-But. The validity of the viscosity measurements was verified for the case of reline (choline chloride:urea, 1:2) containing 25% (*w*:*w*) water, and a good consistency was found with the published data (e.g., 7.29 mPa·s against 7.90 mPa·s as interpolated from Agieienko and Buchner [40], and 7.14 mPa·s from Xie et al. [44] at 25 °C and 3.17 mPa·s against 3.36 mPa·s at 50 °C) [40].

The Vogel–Fulcher–Tammann (VFT) model [45] is used commonly to describe the temperature dependence of the viscosity of aqueous NADES (Equation (1)):

(1) $$\ln\eta =A+\frac{B}{T-T_{0}}$$

The parameters *A*, *B*, and *T*_0_ are usually interpreted merely as empirical constants obtained by fitting a VFT model to the experimental data, as the initial values in the fitting process *A* = −2, *B* = 800 K, and *T*_0_ = 170 K are used by some researchers [45]. The application of the VFT model also gave a good fit of temperature dependence of viscosity of NADES/water systems in this work (Figure S2; the obtained empirical constants *A*, *B*, and *T*_0_ are shown in Table S1, Supplementary Materials). Similarly as in the case of density, a qualitative correlation was found between the viscosities of aqueous solutions of the used HBD [42,43] and the viscosities of the studied NADES/water systems (Figure S3, Supplementary Materials).

In conclusion, the relatively good correlation of the density and viscosity of the studied NADES with the density and viscosity of the aqueous solutions of HBD components used for their preparation (Figures S1 and S3) shows that hydration is an important source of the properties of NADES/water systems. This finding can confirm the conclusion of Mokhtarpour and Shekaari that the interaction between the components (DES/NADES + water) was stronger than the self-interactions between the pure DES/NADES components [46].

### 3.2. Extraction of the Phenolic Compounds of A. millefolium with NADES, and Evaluation of Their Extraction Efficiency

In order to evaluate the extraction efficiency of the selected NADES for the isolation of secondary metabolites (in particular phenolic compounds) from the *A. millefolium* flower, the following conditions were applied: A 75% aqueous solution of NADES and a UAE at the evaluated temperature (50 °C) [23,47,48]. The efficiency of the selected NADES was determined by evaluating the total phenolic content (TPC) and antioxidant capacity (ABTS and FRAP) of the obtained extracts (Table 2). Moreover, the extracts were subjected to UHPLC-MS and HPLC-UV analyses to determine the content (mg g^−1^ DW) of the main bioactive compounds (Table 2). All of these results were also compared with the extracts obtained with conventionally used organic solvents (80% MeOH and 80% EtOH) and ultra pure water (H_2_O).

As shown in Table 2, the highest amounts of phenolic compounds, expressed as TPC value (mg GA g^−1^ DW), were obtained with the lactic acid-based NADES (ChCl-LA, 35.44 ± 2.12 mg GA g^−1^ DW) and urea-based NADES (ChCl-U, 34.98 ± 1.22 mg GA g^−1^ DW), followed closely by ChCl-Prop (31.73 ± 1.17 mg GA g^−1^ DW). All of these NADES were superior to the conventionally used solvents in which the following amounts of TPC were found: 28.92 ± 0.33 mg GA g^−1^ DW (80% EtOH), 27.69 ± 0.49 mg GA g^−1^ DW (80% MeOH), and 21.67 ± 1.30 mg GA g^−1^ DW (water). On the other hand, the lowest TPC was determined in the fructose-based extract (ChCl-Fruc, 12.66 ± 0.75 mg GA g^−1^ DW). The TPC results were well correlated with the previously determined viscosities (Figure 1b) of the studied NADES (lower measured viscosity yielded the higher TPC values), but no correlation was found between the determined TPC and the solvent densities. This is expected, considering that the solvent viscosity affects the mass transport phenomena significantly.

In general, the TPC values of the *A. millefolium* flower obtained in this study were in agreement with the published studies of Becker et al. [6] and Villanueva-Bermejo [3], and were significantly higher compared to the results obtained by Salar Bashi et al. [49]. However, higher TPC values were obtained by Farhadi et al. [2] and Vladić et al. [17]. This discrepancy in the results could be explained by differences in the analyzed plant material (geographical origin, harvest date, and plant part), and the extraction conditions applied (extraction technique, extraction solvent, temperature, etc.).

In terms of the antioxidant activity expressed as mg TE g^−1^ DW (ABTS assay), all of the NADES showed significantly higher values compared to conventional solvents, with the most promising results observed for ChCl-U (51.66 ± 0.59 mg TE g^−1^ DW, Table 2). In general, a good positive correlation was observed between ABTS and TPC, with one exception (ChCl-LA). On the other hand, an extremely high FRAP value (58.52 ± 0.34 mg Fe^2+^ g^−1^ DW) was observed in the same solvent (ChCl-LA) (more than 100% higher than the values for all other solvents). The differences in the results of the antioxidant assays determined by two different tests can probably be explained by the different mechanisms of action. ABTS are hydrogen atom transfer-based assays that quantify the ability to donate hydrogen atoms, whereas FRAP is an electron transfer-based assay that measures antioxidant reduction capacity [50]. It is also worth noting that the reduction of the iron(III)-tripyridyltriazine (Fe^3+^-TPTZ) complex to iron(II)-tripyridyltriazine (Fe^2+^-TPTZ) is a pH-dependent process [36], and among the NADES tested, the lactic acid-based NADES had the lowest pH, resulting in a dramatic increase in the measured FRAP value. These inconsistencies in the results also suggest that it is likely that the NADES solvents themselves affected the antioxidant capacity of the obtained extracts, and a systematic study of the performance with a pure NADES would be advisable.

Although the antioxidant properties of *A. millefolium* have been evaluated previously and reported [7,14,16,17,51], a direct comparison of the results is difficult due to the different ways in which the results are expressed by the authors. For example, Vladić et al. reported an ABTS value of 1730.84 µg TE mL^−1^ under the optimized SWE extraction conditions (temperature 198 °C, extraction time 16.5 min, and without acidifier) [17], while Villalva et al. determined an ABTS value of 0.345 ± 0.002 mmol TE g^−1^ for the extract obtained by supercritical anti-solvent fractionation (SAF) [7]. For the FRAP assay, Tadić et al. reported about 1.26 ± 0.07 µmol Fe^2+^ g^−1^ [14].

**Figure 2:**
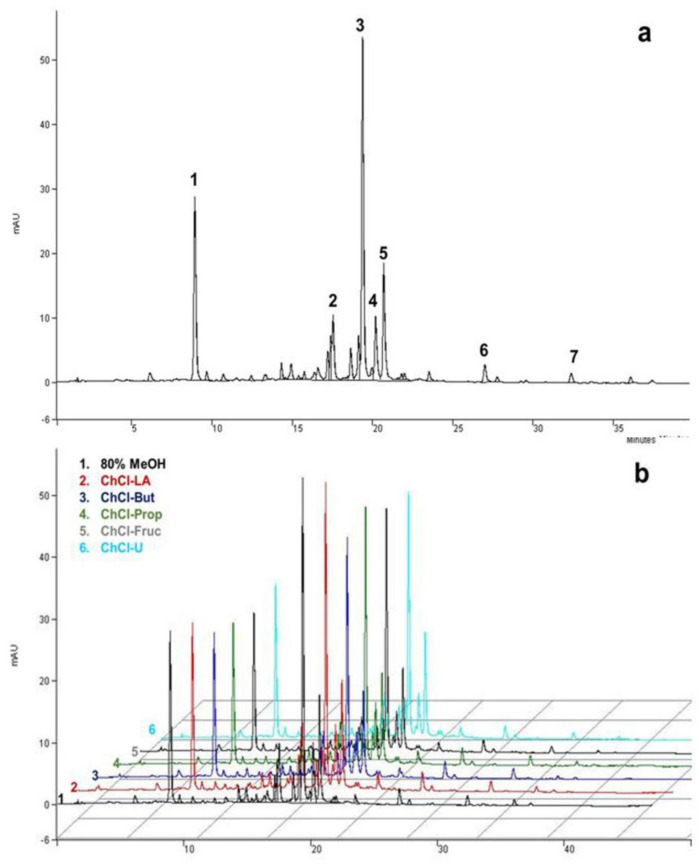
Typical HPLC-UV chromatograms of *A. millefolium* extracts recorded at 325 nm under optimal chromatographic conditions: (**a**) methanolic; (**b**) compression between methanolic and NADES extracts: (1) chlorogenic acid; (2) luteolin-7-*O*-glucoside; (3) DCQA isomer I; (4) apigenin-7-*O*-glucoside; (5) DCQA isomer II; (6) luteolin; (7) apigenin.

Chemical characterization of MeOH, EtOH, or aqueous *A. millefolium* extracts confirmed the presence of various bioactive compounds, dominated by chlorogenic acid, isomers of dicaffeoylquinic acids, and flavonoids [7,14,17,51]. In our particular case, seven different phenolic compounds were identified by HPLC-MS and HPLC-UV, quantified in all of the obtained extracts (Figure 2 and Table 2). The compounds were identified by comparing their relative retention times and mass spectra with the data available in the literature [14,52], while four of them (chlorogenic acid, apigenin-7-*O*-glucoside, luteolin, and apigenin) were additionally confirmed by commercially available standards.

Phenolic acids (chlorogenic acid and two isomers of DCQA) were determined to be the most abundant phenolic compounds in yarrow extracts, with no statistical differences (Table 2) in extraction yield between the NADES and organic solvents tested, while water showed a slightly lower content of DCQA isomers (Table 2). Similar results were also observed in our previous study, where NADES also showed a comparable extraction capacity for phenolic acid from a *Helichrysum arenarium* L. sample as the investigated organic solvents [23]. However, in the study by Peng et al., a slightly superior extraction performance for polyalcohol-based NADES over the others tested was observed for the selective extraction of five DCQA isomers from *Lonicerae Japonicae Flos* [24]. Compared to the other studies, the content of chlorogenic acid obtained in this study (2.65–3.29 mg g^−1^ DW) was slightly higher. Namely, Georgieva et al. determined a chlorogenic acid content of 0.78 mg g^−1^ DW [12], while Tadić et al. reported content of 0.28 mg g^−1^ DW [14]. Vitalini et al. reported two DCQA isomers that were not quantified [8], while Salomon et al. confirmed the presence of three DCQA isomers in the acetone/water mixture, *N*-butanol, and ethanol fractions [51].

In the case of luteolin-7-*O*-glucoside, the highest contents were found in the extracts of ChCl-LA (0.51 ± 0.07 mg g^−1^ DW) and ChCl-Prop (0.51 ± 0.03 mg g^−1^ DW), which were in the range of contents found in the extracts obtained with organic solvents (0.53 ± 0.03 mg g^−1^ DW in 80% EtOH and 0.51 ± 0.02 mg g^−1^ DW in 80% MeOH). The results were in good agreement with those of Tadić et al. [14] (0.66 ± 0.01 mg g^−1^ DW), and were significantly lower than those of Villalva et al., who determined luteolin-7-*O*-glucoside (from 0.39 ± 0.00 mg g^−1^ DW up to 33.2 ± 0.07 mg g^−1^ DW) to be the major phenolic compound in SAF *A. millefolium* extract [7]. A similar trend in extraction efficiency was also observed for luteolin aglicone (Table 2).

ChCl-Prop was proven to be the most promising NADES for the extraction of apigenin-7-*O*-glucoside (1.23 ± 0.02 mg g^−1^ DW), followed by ChCl-LA (1.07 ± 0.18 mg g^−1^ DW), with no statistical differences observed from the results obtained with organic solvents (Table 2). These results are in good agreement with the previously published studies, in which polyalcohol-based NADES were postulated as the most promising NADES solvents for the extraction of flavones for various plant materials [23,53]. The obtained values for apigenin-7-*O*-glucoside were in the range of contents previously reported by Dias et al. ([15] 1.43 mg g^−1^ DW) and Tadić et al. ([14] 1.85 mg g^−1^ DW). On the other hand, apigenin aglicone was quantified only in the ChCl-Prop and methanolic extracts, whereas concentrations below the limit of quantification (LOQ) were detected in the other solvents (Table 2).

From the results evaluated so far, it can generally be concluded that the viscosity and not the density of NADES affected the extraction efficiency. Accordingly, the highest contents of bioactive compounds extracted from *A. millefolium* were found in the lactic acid-based NADES, followed by two polyalcohol-based NADES. However, the differences in the contents were not dramatic, because the extraction process was carried out under an elevated temperature (50 °C), where all solvents had comparable dynamic viscosities (Figure 1b).

### 3.3. Antibacterial and Antifungal Activity of the Obtained Extracts

In addition, the antibacterial and antifungal activity was determined of different *A. millefolium* extracts on four bacterial cultures (*Staphylococcus aureus*, *Bacillus cereus*, *Escherichia coli*, and *Pseudomonas aeruginosa*) and two yeast strains *Candida albicans*. The solvents (MeOH, ChCl-LA, ChCl-But, ChCl-Prop, ChCl-Fruc, and ChCl-U) were tested in parallel as a positive control at the concentrations used for the extract preparations.

The results obtained are presented systematically in Table 3 and Table 4. The UAE extract obtained with ChCl-LA had the lowest MIC and MBC/MFC against all of the microorganisms, with an MIC ranging from 0.05 mg mL^−1^ (for *Bacillus cereus* and *Pseudomonas aeruginosa*) to 0.80 mg mL^−1^ for the yeasts (Table 4). However, the MIC and MBC of the extract were the same as the MIC and MBC (%, *v*:*v*) of the pure solvent (ChCl-LA/water; 75%:25%; *w*:*w*). On the other hand, the extract prepared with ultra pure H_2_O had the weakest inhibitory activity with MIC, and an MBC/MFC higher than 3.2 mg mL^−1^. The 80% EtOH extract (1.6 mg mL^−1^ for all bacteria) and urea-based NADES (ChCl-U, 1.2 mg mL^−1^ for all bacteria) had the most pronounced activity with the lowest MIC. The MBC was different and slightly lower for Gram-negative bacteria for both extracts. Other extracts tested showed an inhibitory activity on the growth of all bacteria with an MIC (mg mL^−1^) generally equal to the MIC (%, *v*:*v*) of the solvents used for their preparation (positive control).

The antifungal activity of the *A. millefolium* extracts studied was similar (Table 4). Four extracts (80% EtOH, 80% MeOH, ChCl-But, and ChCl-Prop) had a slightly lower MIC (1.6–2.0 mg mL^−1^) than the MIC (%, *v*:*v*) of the used solvents. The MFC for these extracts and the MIC and MFC of the other extracts were consistent with the positive control. The H_2_O extract had no effect, even at the highest concentrations. The inhibitory effect of MeOH and EtOH on microorganisms has been confirmed already [54]. On the other hand, data on the inhibitory effect of DES/NADES on microorganisms are quite variable. For example, Hayyan et al. found that DES based on choline chloride (with ethylene glycol, triethylene glycol, and urea) has no antibacterial effect [55], while the same group of researchers reported a mild inhibitory effect of DES based on organic acid [56]. However, many authors have proven the opposite, and confirmed the inhibitory effect of DES/NADES on microorganisms [21,57,58]. In the current study, Jurić et al. evaluated the antimicrobial properties of *Mentha piperita* NADES extracts, and confirmed that all NADES showed an inhibition of bacterial growth at a concentration level lower than 70% EtOH [59]. In our study, the increased inhibitory activity of ChCl-LA and its extract can be associated with the high content of organic acid (lactic acid) [19,56], but also with the higher content of bioactive compounds compared to the other tested solvents (Table 2).

According to the literature, MIC concentrations of *A. millefolium* extracts ranged from a few mg mL^−1^ to 250 mg mL^−1^ or even more, and the inhibition zones also varied [9,10,11]. Consequently, a direct comparison of the numerical values is difficult, as the antimicrobial activity of the extracts is highly dependent on the type of solvent in which the extraction was performed, the part of the plant used for extraction, and the area from which the plant originated.

In antimicrobial testing using the disk diffusion method, extracts of *A. millefolium* were used at concentrations of 100% and 50%, while solvents were tested at concentrations of 80% and 40% for MeOH and EtOH, and 100% and 50% for NADES. These concentrations had no antimicrobial effect, except for ChCl-LA and its corresponding extract (Table 5). The antimicrobial activity of the extract of ChCl-LA, measured as the zone of inhibition, was slightly different from the antimicrobial activity of the pure solvent (Table 5). As the initial concentration (100%) of the extract in ChCl-LA was 20 mg mL^−1^, there was a possibility of interaction between the extract and the solvent that cancelled their effect. However, it was not so simple, because there was a possibility that the extract raised the pH, which is degraded by lactic acid, and that some components of the extract had an antimicrobial activity that cancelled each other. It is also interesting to note that different extracts of *A. millefolium* (80% EtOH, 80% MeOH, ChCl-But, ChCl-Prop, ChCl-Fruc, and ChCl-U) did not show an antimicrobial activity, unlike the MIC and MBC/MFC methods tested by the disk diffusion method (Table 4).

MeOH and EtOH (80%) evaporate rapidly at incubation temperatures, so the active components of the extract did not diffuse into the substrate. On the other hand, NADES in the composite molar ratio probably diffused less into the agar medium, resulting in the absence of an inhibition zone [21,60] (Figures S4 and S5).

Various laboratory methods can be used to evaluate or screen the in vitro antimicrobial activity of an extract or pure compound. One of the most commonly used methods is the use of solid culture media in plates with disks, in which NADES is embedded (antibiogram-like assay) [61]. On the other hand, the major drawback of this method is an important limitation due to the high density and viscosity that characterize most NADES [62]. The high density and viscosity limit the diffusion of NADES from the disk to the surrounding environment, leading to results that may not reflect the actual interaction between NADES and the cells. The use of the liquid medium minimizes these negative effects, especially in cases where NADES is particularly dense or has a high viscosity [63]. However, in this study, the second lowest viscosity was found for lactic acid-based NADES (ChCl-LA, 4.24 mPa·s), which allowed it to diffuse easily from the disk into the agar. In addition, it should be noted that the overall lower susceptibility observed for *C. albicans* may be due to the lower incubation temperature for this microorganism (30 °C) compared to that used for the bacterial strains (37 °C), which affected the diffusion of the compounds/NADES formulations through the solid media, along with the lower viscosity at a higher temperature [59].

## 4. Conclusions

In this study, a new, more environmentally friendly and effective extraction method based on ultrasound-assisted extraction (UAE) and natural deep eutectic solvents (NADES) was applied for the isolation of bioactive compounds from yarrow flowers (*A. millefolium* L.). Based on the observed results, it can be concluded that lactic acid-based NADES (ChCl-LA) is a promising solvent, with extraction results (expressed as TPC) higher than those obtained with 80% MeOH, 80% EtOH, and water. In addition, the antioxidant capacity of the NADES extracts was higher in most cases (an exception was the fructose-based NADES). The results obtained for the TPC and antioxidant tests were in good negative correlation with the measured viscosities (lower solvent viscosity resulted in a higher extraction capacity), while the solvent density had no significant effect on the extraction capacity. Moreover, the UAE extract obtained with ChCl-LA had the lowest MIC and MBC/MFC against all microorganisms, and, on the other hand, the extract prepared with ultrapure H_2_O had the weakest inhibitory activity against all microorganisms. In the disk diffusion method, only the extract of ChCl-LA showed a antimicrobial activity, while the other extracts had no effect and there was no inhibition zone.

## Figures and Tables

**Table 1:** Composition and the molar ratio of the NADES evaluated in this study.

Abbreviation	Compounds	Molar Ratio
ChCl-LA	Choline chloride:lactic acid	1:2
ChCl-But	Choline chloride:1,4-butanediol	1:2
ChCl-Prop	Choline chloride:1,2-propanediol	1:2
ChCl-Fruc	Choline chloride:fructose:water	2:1:1
ChCl-U	Choline chloride:urea	2:1

**Table 2:** The content of individual phenolic compounds (mg g^−1^ DW) of *A. millefolium* in the extracts obtained with five different NADES (25% water) compared with 80% EtOH, 80% MeOH, and water. The results are expressed as mean value ± standard deviation.

t*_R_*	Compound	80% EtOH	80% MeOH	H_2_O	ChCl-LA	ChCl-But	ChCl-Prop	ChCl-Fruc	ChCl-U
9.03	^1^ Chlorogenic acid	3.29 ± 0.01 ^a^	3.15 ± 0.13 ^a^	2.88 ± 0.18 ^a^	2.97 ± 0.12 ^a^	2.65 ± 0.05 ^a^	2.78 ± 0.20 ^a^	2.85 ± 0.45 ^a^	2.90 ± 0.10 ^a^
17.63	^2^ Luteolin-7-*O*-glucoside	0.53 ± 0.03 ^b^	0.51 ± 0.02 ^b^	0.28 ± 0.04 ^a^	0.51 ± 0.07 ^b^	0.40 ± 0.08 ^a,b^	0.51 ± 0.03 ^b^	0.34 ± 0.04 ^a,b^	0.39 ± 0.06 ^a,b^
19.25	^3^ DCQA isomer I	6.45 ± 0.05 ^b^	6.29 ± 0.31 ^b^	3.83 ± 0.60 ^a^	5.79 ± 0.46 ^b^	4.98 ± 0.11 ^b^	5.35 ± 0.18 ^b^	5.38 ± 0.80 ^b^	5.19 ± 0.32 ^b^
20.25	^4^ Apigenin-7-*O*-glucoside	1.14 ± 0.01 ^c,d^	1.17 ± 0.09 ^c,d^	0.24 ± 0.00 ^a^	1.07 ± 0.18 ^c^.^d^	1.02 ± 0.11 ^c,d^	1.23 ± 0.02 ^d^	0.77 ± 0.13 ^b^	0.86 ± 0.11 ^b,c^
20.72	^3^ DCQA isomer II	2.35 ± 0.01 ^b^	2.28 ± 0.03 ^b^	1.31 ± 0.19 ^a^	2.21 ± 0.13 ^b^	2.05 ± 0.10 ^b^	2.16 ± 0.06 ^b^	1.89 ± 0.32 ^b^	2.13 ± 0.10 ^b^
27.08	^2^ Luteolin	0.12 ± 0.01 ^b^	0.10 ± 0.01 ^b^	<LOQ	0.14 ± 0.00 ^b^	0.13 ± 0.03 ^b^	0.11 ± 0.00 ^b^	<LOQ	<LOQ
32.46	^5^ Apigenin	0.04 ± 0.01 ^b^	<LOQ	<LOQ	<LOQ	>LOQ	0.04 ± 0.01 ^b^	<LOQ	<LOQ
* TPC		28.92 ± 0.33 ^c^	27.69 ± 0.49 ^c^	21.67 ± 1.30 ^b^	35.44 ± 2.12 ^d^	28.82 ± 2.06 ^c^	31.73 ± 1.17 ^c,d^	12.66 ± 0.75 ^a^	34.98 ± 1.22 ^d^
^$^ ABTS		40.95 ± 0.57 ^a,b,c^	40.51 ± 1.63 ^a^	42.75 ± 2.23 ^a,b,c^	41.48 ± 1.33 ^a,b^	46.89 ± 1.82 ^b,c,d^	46.70 ± 0.52 ^b,c,d^	47.75 ± 2.76 ^c,d^	51.66 ± 0.59 ^d^
ᵜ FRAP		22.44 ± 0.20 ^a^	22.50 ± 0.57 ^a^	18.84 ± 1.81 ^a^	58.52 ± 0.34 ^b^	24.71 ± 0.65 ^a^	24.28 ± 0.64 ^a^	22.87 ± 3.77 ^a^	21.98 ± 0.64 ^a^

^a,b,c,d^ Different superscripts for each compound in the same row denoted significant differences between the solvents tested with a 95% confidence level (*p* < 0.05) according to the Student–Newman–Keuls (S-N-K) test. Different superscript numbers indicate the external standard used for quantification: ^1^ Chlorogenic acid; ^2^ luteolin; ^3^ 1,5-dicaffeoylquinic acid; ^4^ apigenin-7-*O*-glucoside; ^5^ apigenin; LOQ-limit of quantification; * total phenolic content (TPC) expressed as milligrams of gallic acid equivalent per gram of dry weight (mg GA g^−1^ DW); ^$^ antiradical capacity of the extracts, expressed as milligrams of the trolox equivalent per gram of dry weight (mg TE g^−1^ DW); ᵜ antioxidant activity of extracts, expressed as milligrams of the Fe^2+^ ion equivalent per gram dry weight (mg Fe^2+^ g^−1^ DW).

**Table 3:** Antibacterial (MIC and MBC in % *v*/*v*) and antifungal (MIC and MFC in % *v*/*v*) activity of used solvents.

Solvent	Antibacterial Activity								Antifungal Activity			
	*S. aureus*		*B. cereus*		*E. coli*		*P. aeruginosa*		*Candida albicans* ATCC 10231		*Candida albicans* Isolate	
	MIC	MBC	MIC	MBC	MIC	MBC	MIC	MBC	MIC	MFC	MIC	MFC
80% EtOH	10	12	8	>16	10	14	10	10	8	12	8	12
80% MeOH	10	12	10	>16	12	16	8	12	8	14	8	14
Ultra pure H_2_O	0	0	0	0	0	0	0	0	/	/	/	/
ChCl-LA *	0.5	1	0.25	2	0.5	1	0.25	1	4	4	4	6
ChCl-But *	14	14	14	>16	14	16	10	>16	10	14	10	14
ChCl-Prop *	14	14	14	>16	14	16	12	>16	12	14	12	14
ChCl-Fruc *	14	16	16	>16	14	>16	14	>16	12	14	12	14
ChCl-U *	10	12	10	16	10	14	10	14	10	14	10	12

* All solvents contains 25% of ultra pure H_2_O (NADES:H_2_O; 75:25; *w*:*w*).

**Table 4:** Antibacterial (MIC and MBC in mg mL^−1^) and antifungal (MIC and MFC in mg mL^−1^) activity of *A. millefolium* extracts obtained after UAE at an elevated (50 °C).

Sample	Antibacterial Activity								Antifungal Activity			
	*S. aureus*		*B. cereus*		*E. coli*		*P. aeruginosa*		*Candida albicans* ATCC 10231		*Candida albicans* Isolate	
	MIC	MBC	MIC	MBC	MIC	MBC	MIC	MBC	MIC	MFC	MIC	MFC
80% EtOH	1.6 (6.4) *	2.0 (8.0)	1.6 (6.4)	2.4 (9.6)	1.6 (6.4)	2.0 (8.0)	1.6 (6.4)	1.6 (6.4)	1.6 (6.4)	2.0 (8.0)	1.6 (6.4)	2.0 (8.0)
80% MeOH	2.4 (9.6)	2.4 (9.6)	2.4 (9.6)	2.8 (11.2)	2.4 (9.6)	2.8 (11.2)	2.0 (8.0)	2.4 (9.6)	1.6 (6.4)	2.4 (9.6)	2.0 (8.0)	>2.4 (>9.6)
Ultra pure H_2_O	>3.2	>3.2	>3.2	>3.2	>3.2	>3.2	>3.2	>3.2	>3.2	>3.2	>3.2	>3.2
ChCl-LA	0.1 (0.5)	0.2 (1.0)	>0.1 (0.3)	0.2 (1.0)	0.1 (0.5)	0.2 (1.0)	>0.1 (0.23)	0.1 (0.5)	0.8 (4.0)	1.2 (6.0)	0.8 (4.0)	1.2 (6.0)
ChCl-But	2.8 (14.0)	2.8 (14.0)	2.4 (12.0)	>3.2 (>16.0)	2.4 (12.0)	>3.2 (>16.0)	2.0 (10.0)	>3.2 (>16.0)	1.6 (8.0)	2.4 (12.0)	1.6 (8.0)	2.4 (12.0)
ChCl-Prop	2.8 (14.0)	2.8 (14.0)	2.8 (14.0)	>3.2 (>16.0)	2.4 (12.0)	>3.2 (>16.0)	2.0 (10.0)	3.2 (16.0)	1.6 (8.0)	2.4 (12.0)	2.0 (10.0)	2.4 (12.0)
ChCl-Fruc	2.8 (14.0)	3.2 (16.0)	3.2 (16.0)	>3.2 (>16.0)	3.2 (16.0)	>3.2 (>16.0)	2.4 (12.0)	>3.2 (>16.0)	2.4 (12.0)	2.8 (14.0)	2.4 (12.0)	2.8 (14.0)
ChCl-U	1.2 (6.0)	2.4 (12.0)	1.2 (6.0)	3.2 (16.0)	1.2 (6.0)	1.6 (8.0)	1.2 (6.0)	2.0 (10.0)	2.0 (10.0)	2.8 (14.0)	2.0 (10.0)	2.8 (14.0)

* Numbers presented in parentheses are MIC or MBC or MFC (% *v*/*v*) of solvents used for the preparation of extracts, which correspond to the MIC or MBC or MFC (mg mL^−1^).

**Table 5:** Antibacterial and antifungal activity of pure ChCl-LA and ChCl-LA extract (two different concentrations) in comparison with the selected antibiotics. The results are expressed as the zone of inhibition (Mean value ± Standard Deviation) in mm.

	Pure Solvent		UAE Extract		E 15 µg	G 10 µg	A 10 µg	C 5 µg	Ny 100 U.	FlU 100 µg
	ChCl-LA 100%	ChCl-LA 50%	ChCl-LA 100%	ChCl-LA 50%						
Bacteria										
*S. aureus*	28.67 ± 0.58 ^b^	21.67 ± 0.53 ^a^	27.17 ± 1.17 ^b^	20.83 ± 0.75 ^a^	28.80 ± 2.05 ^b^	29.20 ± 2.17 ^b^	34.80 ± 1.79 ^c^	31.20 ± 1.64 ^b^	n.a.	n.a.
*B. cereus*	26.67 ± 0.58 ^b^	23.17 ± 0.29 ^b^	27.17 ± 0.63 ^b^	21.92 ± 0.20 ^b^	28.00 ±2.35 ^b^	24.80 ± 1.48	12.40 ± 3.21 ^a^	29.20 ± 1.64 ^b^	n.a.	n.a.
*E. coli*	26.67 ± 0.58 ^b^	20.50 ± 0.50 ^a^	25.59 ± 1.96 ^b^	19.67 ± 0.52 ^a^	n.d	22.60 ± 1.52 ^a,b^	23.00 ± 1.22 ^a,b^	33.40 ± 1.52 ^c^	n.a.	n.a.
*P. aeruginosa*	29.66 ± 0.58 ^b^	21.83 ± 0.29 ^a^	31.34 ± 0.41 ^b^	21.83 ± 1.29 ^a^	n.d	21.40 ± 1.14 ^a^	0	31.60 ± 1.67 ^b^	n.a.	n.a.
Yeasts										
*Candida albicans* ATTC 10231	8.33 ± 0.58 ^a^	6.5 ± 0.00 ^a^	8.42 ± 0.35 ^a^	6.33 ± 0.27 ^a^	n.a.	n.a.	n.a.	n.a.	25.75 ± 2.99 ^b^	n.d
*Candida albicans* isolate	6.55 ± 0.00 ^a^	n.d.	6.67 ± 0.29 ^a^	n.d.	n.a.	n.a.	n.a.	n.a.	25.50 ± 3.11 ^b^	n.d

^a,b,c^ Different superscripts for each bacteria/yeast tested in the same row denote significant differences between the solvents/extracts/antibiotics tested at a 95% confidence level (*p* < 0.05) according to the Student–Newman–Keuls (S-N-K) test; E—Erythromycin; G—Gentamicin; A—Ampicillin; C—Ciprofloxacin; Ny—Nystatin; Flu—Fluconazole; n.a.—not applicable; n.d.—not determined.

## Data Availability

Data are contained within the article and Supplementary Material.

## Supplementary Materials

The following supporting information can be downloaded at: https://www.mdpi.com/article/10.3390/antiox11040724/s1. Figure S1: Correlation between the density of the studied NADES (the point for choline chloride/fructose:water DES is omitted) and the density of aqueous solutions containing hydrogen bond donor (HBD) components (1,4-butanediol, 1,2-propanediol, lactic acid, and urea, respectively) of comparable concentrations. The data refer to the temperature 25 °C (298.15 K). Figure S2: Temperature dependence of η (logarithmic scale) of the studied NADES containing 25% water (*w*:*w*) as described by the VFT model (broken lines) in comparison with the experimental data (points). Figure S3: Correlation between the viscosity of the studied NADES (the point for choline chloride:fructose:water NADES is omitted) and the viscosity of aqueous solutions containing HBD components (1,4-butanediol, 1,2-propanediol, lactic acid, and urea, respectively) of comparable concentrations. The data refer to the temperature 25 °C (298.15 K). Figure S4: Antifungal activity (MIC) of urea-based NADES (ChCl-U) on *Candida albicans* ATCC 10231 (C) and *Candida albicans* isolate (Ci). Figure S5: Antimicrobial activity of choline chloride-urea based NADES (ChCl-U; pure solvent) and ChCl-U extract on *Staphylococcus aureus* ATCC 25923 (disk diffusion method). Table S1: Values of empirical coefficients from the Vogel–Fulcher–Tammann (VFT) equation (Equation (1) in the manuscript) obtained by fitting the VFT model to experimental data for the studied NADES. The temperature range of fitting was 5–60 °C (278.15–333.15 K).
