# Supplementary material for: Extraction of Bioactive Metabolites from Achillea millefolium L. with Choline Chloride Based Natural Deep Eutectic Solvents: A Study of the Antioxidant and Antimicrobial Activity
Source: Antioxidants (Basel). 2022 Apr 6;11(4):724. doi: 10.3390/antiox11040724 (PMC9027353; doi:10.3390/antiox11040724)
Supplement: Supplementary file 1 [file antioxidants-11-00724-s001.zip › antioxidants-1603004-supplementary-done.pdf]

antioxidants

MDPI

SUPPLEMENTARY MATERIAL

Extraction of bioactive metabolites from *Achillea Millefolium* L. with choline chloride based natural deep eutectic solvents; study of antioxidant and antimicrobial activity

Milena Ivanović[2], Dragana Grujić[3], Janez Cerar[1], Maša Islamčević Razboršek[2], Ljiljana Topalić-Trivunović[3], Aleksandar Savić[3], Drago Kočar[1], Mitja Kolar[1,*]

[1]University of Ljubljana, Faculty of Chemistry and Chemical Technology, Večna pot 113, SI-1000 Ljubljana, Slovenia
[2]University of Maribor, Faculty of Chemistry and Chemical Engineering, Smetanova ulica 17, SI-2000 Maribor, Slovenia
[3]University of Banja Luka, Faculty of Technology, Vojvode Stepe Stepanovića 73, 78000 Banja Luka, Bosnia and Herzegovina

Corresponding author: mitja.kolar@fkkt.uni-lj.si (Tel.: +386-1-479-8694)

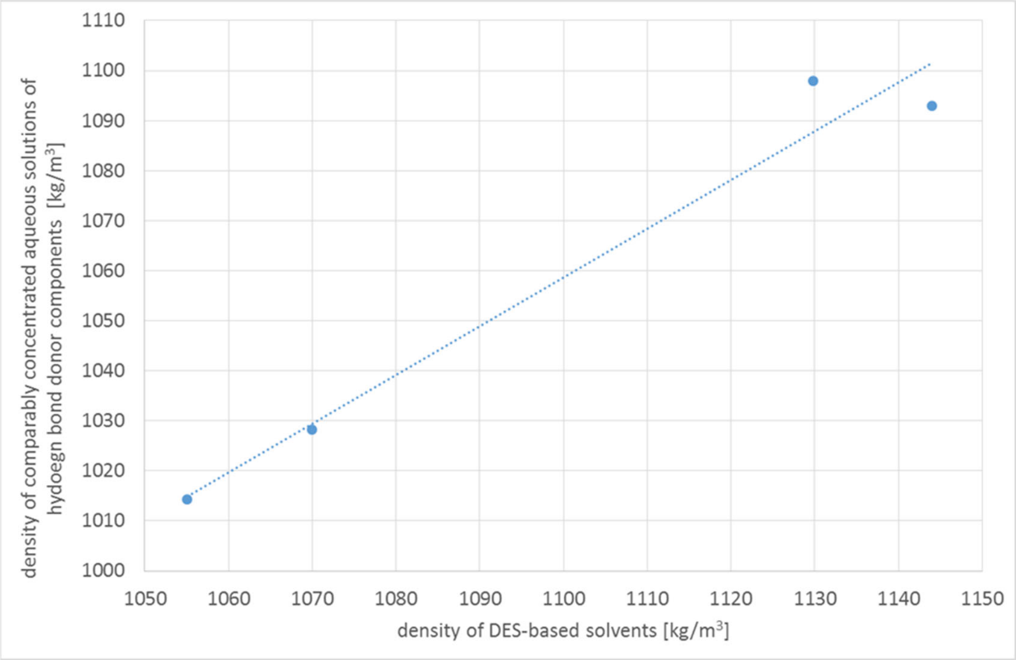

Figure S1. Correlation between the density of the studied NADES (the point for choline chloride:fructose:water DES is omitted) and the density of aqueous solutions containing hydrogen bond donor (HBD) components (1,4-butanediol, 1,2-propanediol, lactic acid, and urea, respectively) of comparable concentrations. The data refer to the temperature 25 °C (298.15 K).

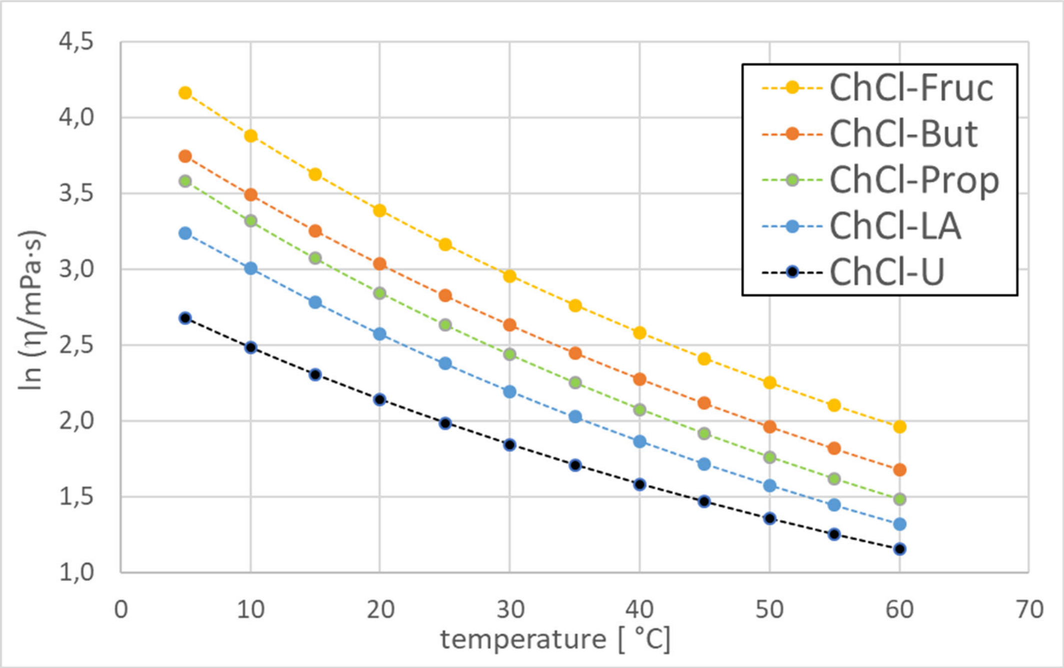

Figure S2. Temperature dependence of η (logarithmic scale) of the studied NADES containing 25% water (w:w) as described by the VFT model (broken lines) in comparison with the experimental data (points).

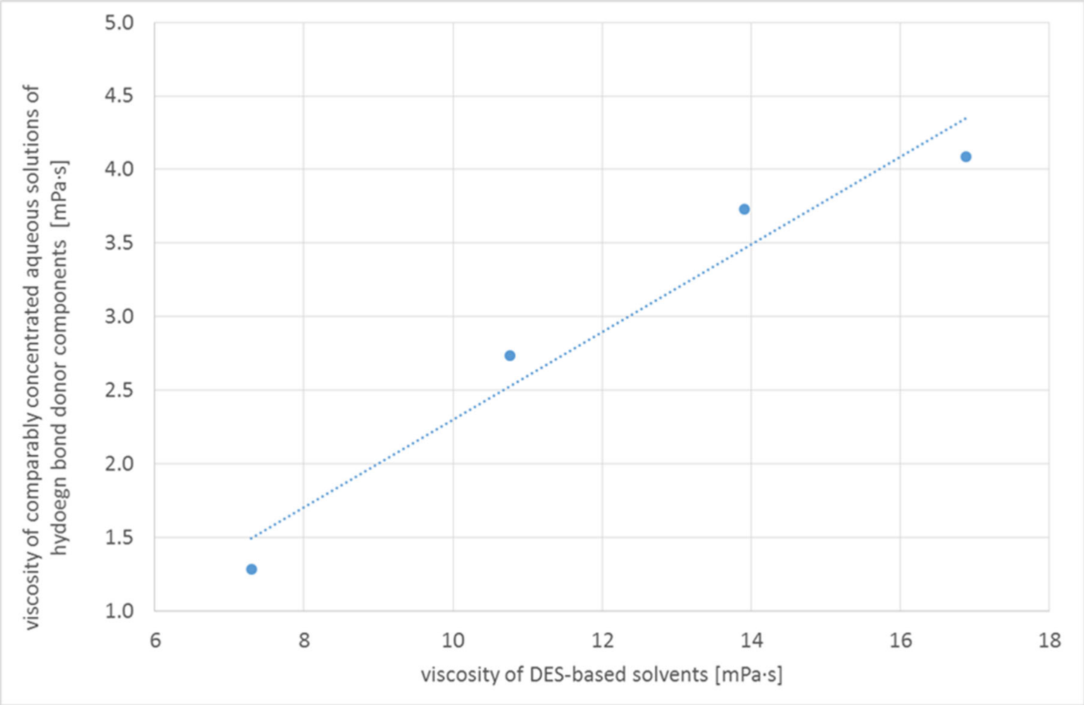

Figure S3. Correlation between the viscosity of the studied NADES (the point for choline chloride:fructose:water NADES is omitted) and the viscosity of aqueous solutions containing HBD components (1,4-butanediol, 1,2-propanediol, lactic acid, and urea, respectively) of comparable concentrations. The data refer to the temperature 25 °C (298.15 K).

Concentration (%, v:v)	12 %	10%	8%	6%
C. albicans isolate	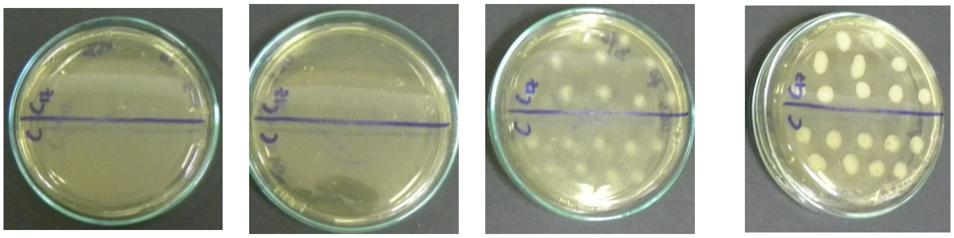			
C. albicans ATCC 10231				

Figure S4. Antifungal activity (MIC) of urea-based NADES (ChCl-U) on *Candida albicans* ATCC 10231 (C) and *Candida albicans isolate* (Ci).

	ChCl-U (pure solvent)	ChCl-U (extract)
100%	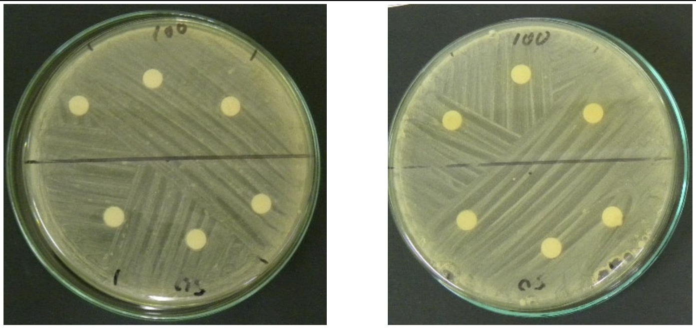	
50%		

Figure S5. Antimicrobial activity of choline chloride-urea besed NADES (ChCl-U; pure solvent) and ChCl-U extract on *Staphylococcus aureus* ATCC 25923 (disk diffusion method).

Table S1. Values of empirical coefficients from the Vogel-Fulcher-Tammann (VFT) equation (eq. 1 in the manuscript) obtained by fitting the VFT model to experimental data for the studied NADES. The temperature range of fitting was 5-60 °C (278.15-333.15 K).

NADES	ln (A/η°)	B [K]	T_0 [K]	η range (mPa·s)
ChCl-LA	-3.167	821.09	150.03	25.5–3.7
ChCl-But	-3.419	971.92	142.52	42.4–25.4
ChCl-Prop	-3.259	849.02	154.03	35.9–4.4
ChCl-Fruc	-2.946	870.89	155.61	64.1–7.1
ChCl-U	-2.279	612.87	154.48	14.5–3.2
